# Purification and characterization of cold-adapted and salt-tolerant dextranase from *Cellulosimicrobium* sp. THN1 and its potential application for treatment of dental plaque

**DOI:** 10.3389/fmicb.2022.1012957

**Published:** 2022-11-11

**Authors:** Linxiang Xu, Yan Zhang, Nannan Liu, Zhen Wei, Zhen Wang, Yonghua Wang, Shujun Wang

**Affiliations:** ^1^Co-Innovation Center of Jiangsu Marine Bio-industry Technology, Jiangsu Key Laboratory of Marine Bioresources and Environment, Jiangsu Ocean University, Lianyungang, China; ^2^School of Food Science and Engineering, South China University of Technology, Guangzhou, China; ^3^Jiangsu Institute of Marine Resources Development, Jiangsu Ocean University, Lianyungang, China; ^4^School of Marine Science and Fisheries, Jiangsu Ocean University, Lianyungang, China

**Keywords:** salt-tolerant, cold-adapted, marine dextranase, dental plaque, isomalto-oligosaccharides_5_

## Abstract

The cold-adapted and/or salt-tolerant enzymes from marine microorganisms were confirmed to be meritorious tools to enhance the efficiency of biocatalysis in industrial biotechnology. We purified and characterized a dextranase CeDex from the marine bacterium *Cellulosimicrobium* sp. THN1. CeDex acted in alkaline pHs (7.5–8.5) and a broad temperature range (10–50°C) with sufficient pH stability and thermostability. Remarkably, CeDex retained approximately 40% of its maximal activities at 4°C and increased its activity to 150% in 4 M NaCl, displaying prominently cold adaptation and salt tolerance. Moreover, CeDex was greatly stimulated by Mg^2+^, Na^+^, Ba^2+^, Ca^2+^ and Sr^2+^, and sugarcane juice always contains K^+^, Ca^2+^, Mg^2+^ and Na^+^, so CeDex will be suitable for removing dextran in the sugar industry. The main hydrolysate of CeDex was isomaltotriose, accompanied by isomaltotetraose, long-chain IOMs, and a small amount of isomaltose. The amino acid sequence of CeDex was identified from the THN1 genomic sequence by Nano LC–MS/MS and classified into the GH49 family. Notably, CeDex could prevent the formation of *Streptococcus mutans* biofilm and disassemble existing biofilms at 10 U/ml concentration and would have great potential to defeat biofilm-related dental caries.

## Introduction

Dextran is a water-soluble glucan (10^5^–10^7^ Da) with mainly α-1,6-glycosidic linkages, generally synthesized from sucrose by dental caries pathogens and microorganisms polluting sugarcane and beet juice (Bashari, 2019; Martínez et al., 2021; Tanoue Batista et al., 2021). Dextranase (E.C.3.2.1.11) specially hydrolyzes the α-1,6-glucosidic linkages in dextran and generates low-molecular-weight (LMW) dextran, isomalto-oligosaccharide (IMO) and/or glucose (Khalikova et al., 2005). LMW dextran with antithrombotic activity is a blood substitute in emergencies and can be used to synthesize iron dextran to treat severe anemia (Khalikova et al., 2005). As one of the promising prebiotic candidates, IMOs modulate the composition of the intestinal microbiome and mediate health-promoting benefits (Sorndech, 2018; Huang et al., 2020; Park et al., 2021). In addition, dextranase can effectively restrain and eliminate dental plaque biofilm, which is greatly significant for the inhibition and treatment of dental caries and the exploitation of the dental caries vaccine (Walker et al., 2016; Pleszczyńska et al., 2017).

Dextranases were from plentiful microbes, such as *Penicillium*, *Aspergillus*, *Chatomium*, *Lipomyces*, *Sporothrix*, *Streptococcus*, *Bifidobacterium*, *Arthrobacter*, *Pseudomonas*, and others (Khalikova et al., 2005; Ren et al., 2018). Dextranases are classified into glycoside hydrolase (GH) 49, 66, 27 and 31 families based on protein structures and sequence similarity (Okazawa et al., 2015; Ren et al., 2019; Tsutsumi et al., 2019). Fungal dextranases often had high activity (generally more than 100 U/mg), but they possessed poor thermal stability and were also unstable under alkaline conditions (Huang et al., 2019). Their optimistic temperature was frequently higher than 50°C, and the optimistic pH was lower than 7 (Khalikova et al., 2005; Zhao et al., 2020; Pittrof et al., 2021). The marine dextranases with cold adaptation, thermostability and/or high catalytic activity in alkaline conditions had been reported and they had an advantage in removing plaque and preventing dental (Ren et al., 2018; Lai et al., 2019; Deng et al., 2020). The dextranases from marine bacteria, such as *Cellulosimicrobium* sp. PX02, *Arthrobacter oxydans* KQ11, *Catenovulum agarivorans* MNH15 and *Bacillus aquimaris* S5, have the characteristics of alkali resistance but did not possess cold-adaptation and salt tolerance (Lai et al., 2019; Liu et al., 2019; Ning et al., 2021). The concentration of PX02 dextranase needed to reach 15 U/ml to remove and inhibit more than 90% of the *S. mutans* biofilms (Ning et al., 2021). The dextranase Aodex from *Arthrobacter oxydans* KQ11 was a mesophilic enzyme (only reacted well at 45–60°C; Liu et al., 2019). When the concentration of Aodex was 6 U/ml, the biofilm inhibition ratio was 90% and the reduction ratio was only 65% (Wang et al., 2016). The dextranase of *Catenovulum agarivorans* MNH15 had an efficient effect on inhibiting the biofilm formed by *S. mutans*, but the SDS and lanric acid, the common reagents in oral hygiene products, had negative effects on enzyme activity (Lai et al., 2019). The dextranase from *Catenovulum* sp. DP03 was cold-adapted and alkali-resistant, some mouthwash ingredients could enhance its activity, and yet its biofilm inhibitory effect was inferior to dextranases from *Arthrobacter oxydans* KQ11 and *Catenovulum agarivorans* MNH15 (Ren et al., 2018; Deng et al., 2020). Many elements influence the activity and stability of dextranase, such as temperature, acid, base, metal ion, surfactant and other components of oral care products (Juntarachot et al., 2020a). It is still challenging to find a suitable and effective dextranase used in oral products because of their poor stability and low activity at medium and low temperatures (Qiu et al., 2016). Therefore, it is highly significant to find a dextranase with high catalytic efficiency at medium and low temperatures and high stability under different types of salts and chemical reagents.

There are a wide variety of microorganisms and other biological resources in marine environments (Kennedy et al., 2011). Because of the low temperature, saline and oligotrophic features of marine environment, marine bacteria evolved several physiological and molecular strategies to adapt to the suffered multiple stresses (Jin et al., 2019). For instance, a unique composition of the cytomembrane decreasing the membrane fluidity and producing compatible solutes (e.g., osmolytes; De Maayer et al., 2014; Collins and Margesin, 2019), ice-binding proteins (Bar Dolev et al., 2016; Vance et al., 2019) and cold-active enzymes (Santiago et al., 2016) all contribute to marine bacteria’s survival in low-temperature condition in the ocean (De Maayer et al., 2014; Mangiagalli and Lotti, 2021). Marine highly saline conditions, especially deep sea, exist salt-tolerant and/or halophiles bacteria (2–5 M NaCl or KCl; Jin et al., 2019). These bacteria balanced their osmotic pressure by importing inorganic ions (K^+^, Na^+^, Cl^−^) or synthesizing abundant specific organic osmoprotectants, and produced salt-active or salt-tolerant enzymes to maintain the basal metabolic rate (Tadeo et al., 2009; Ma et al., 2010; Yin et al., 2015; Jin et al., 2019). Therefore, more and more extremozymes with salt- and pH-tolerance, cold-adaption and thermostability were found in marine bacteria and have potential applications in the extreme conditions of industrial processes (Raddadi et al., 2015; Theerachat et al., 2018; Yang et al., 2018; Karan et al., 2020; Wang et al., 2021).

It is of great importance to explore and utilize marine cold-adapted, salt-tolerant and more stable marine dextranase resources for the dental health industry. This study was pursued to isolate marine dextranase-producing bacteria and obtained new marine dextranases with special characteristics. A cold-adapted and salt-tolerant dextranase from a marine bacterium was identified and characterized to reveal its enzymatic properties, hydrolysis characteristics, protein sequence and 3D structure and its effect on suppressing and removing dental plaque. It provides a new enzymatic tool for plaque removal oral products and will promote the commercial application of marine dextranase.

## Materials and methods

### Materials and chemicals

Blue Dextran 2000 and HiTrap Q FF were purchased from GE Healthcare (Uppsala, Sweden). Different molecular weight dextran and Coomassie brilliant blue G250 were obtained from Solarbio (Beijing, China). Oligosaccharide standards were available from Glycarbo (Osaka, Japan). The protein marker was from Sangon Biotech (Shanghai, China). The other reagents were of analytical grade and purchased from Sinopharm Chemical Reagent Corporation (Shanghai, China).

### Screening and identification of strains

The sea mud samples were collected from Haizhou Bay of Lianyungang to screen dextranase-producing strains by the dilution separation method and the formation of clear zones on the blue dextran plate (Lai et al., 2019). The methods of observing colony morphology and phylogenetic analysis based on 16S rDNA were as previously described (Lai et al., 2019). The 16S rDNA gene sequence of strain THN1 was submitted to NCBI with a GenBank number ON815376.

### Production and purification of dextranase

The strain THN1 was cultured in the medium containing dextran T20 at 5 g, tryptone at 15 g, NaCl at 5 g, bran at 8 g, and aged seawater at 1 l with pH 8.2, and incubated at 30°C for 24 h. The crude enzyme was the supernatant of fermented broth obtained by centrifugation (6,000 rpm, 4°C).

The supernatant was concentrated by the 30,000 NMWC centrifugal filter units (Millipore, Billerica, United States). The saturated ammonium sulfate was added to the concentrated enzyme solution slowly at 4°C. The protein precipitation of different concentrations of ammonium sulfate (20–90%) was collected by centrifugation, dissolved and dialyzed in 20 mM Tris–HCl buffer (pH 7.5) overnight at 4°C. Then the dextranase was purified by HiTrap Q Sepharose FastFlow on AKTA Pure Chromatography system (GE Healthcare, Uppsala, Sweden). The detailed operation referred to Liu et al. (2022). The fractions possessing high dextranase activity were gathered and analyzed by SDS-PAGE (8%).

### Enzymatic characterization

#### Enzyme assay

The dextranase activity was determined by measuring the reduced sugar with a 3,5-dinitrosalicylic acid assay (DNS), with glucose as a standard. The enzyme activity (U) was defined as the amount of enzyme that produces 1 μmol reduced sugars per min under specific conditions. The protein concentration was measured by the BCA protein assay kit (Solarbio, Beijing, China). Unless stated otherwise, the reaction mixture contained 20 mM sodium phosphate buffer (pH 8.0) and 1% (w/w) dextran T20.

#### Effects of temperature and pH on dextranase activity and stability

The optimal temperature of the dextranase was measured at pH 8.0 in temperatures between 4 and 60°C. Thermostability was evaluated by testing the residual enzyme activity after the pre-incubation of the purified CeDex in 20 mM sodium phosphate buffer (pH 8.0) at 30, 40 and 50°C for 1–5 h. The optimal pH was tested at 40°C in 50 mM buffers with different pHs (3.0–9.0), which was determined in four different pH buffer systems. The pH stability was evaluated by measuring the residual activity under the optimal reaction condition after pre-incubation of CeDex in 50 mM buffers in the different pH buffers (3.0–9.0) at 4°C for 16 h.

#### Effects of NaCl on dextranase activity and stability

Determination of the effects of different NaCl concentrations on CeDex: the reaction mixtures with varying concentrations of NaCl (0–5 M) were prepared and reacted under the optimum condition. Determination of NaCl stability of CeDex: the purified CeDex solution with different concentrations of NaCl (0–5 M) was incubated at 4°C for 24 h, and then measured the residual enzyme activity under the optimum condition. The control group was under the same conditions without NaCl treatment.

#### Effects of metal ions and other compounds on dextranase activity

The effects of metal ions and other reagents were valued by testing the enzyme activity of different the CeDex reaction systems with various metal ions and reagents. The reagents included xylitol, sodium fluoride, sodium benzoate, ethanol, sodium dodecyl sulfonate, and lauric acid. The enzyme activity without metal ions or reagents was taken as the control.

#### Dextranase kinetics

The initial reaction velocities (v) of different substrate concentrations (0.1–2.5 mg/ mL) and different Mw dextran (T10, T20, and T70) were calculated under the optimum condition. The kinetic constants *K_m_* and *V_max_* were measured by Lineweaver-Burk plot (Lineweaver and Burk, 1934).

### Hydrolysate analysis

Purified CeDex was incubated with 1% dextran T10, T20, T40, and T70 under the optimum condition for different times (4–24 h). The hydrolysis products were analyzed by -layer chromatography (TLC; aluminum sheet silica gel 60, F254; Merck, Darmstadt, Germany) using the n-propanol/ethyl acetate/acetonitrile/acetic acid/water (5,2,8,1,4.5 by vol.) solvent solution, then the plate was sprayed with the chromogenic reagent (0.2% (w/v) orcinol, 10% (v/v) H_2_SO_4_, and 70% (v/v) ethyl alcohol) followed with incubation at 95°C for 5 min. The standard sugars were glucose, isomaltose and isomaltotriose.

The hydrolysis products of CeDex on the substrate of dextran T20 were identified and quantified by Waters 600 with Waters Sugar-Pak1 (6.5 × 300 mm; Waters, Milford, MA, United States) and a differential refraction detector. The concrete process was performed as previously described (Lai et al., 2019). The peak area was quantified using Empower GPC (Gel Permeation Chromatography) software.

### Amino acid sequence analysis and structure prediction of purified CeDex

The purified CeDex was sequenced by nano LC–MS/MS in Shanghai Applied Protein Technology Co. Ltd. (Shanghai, China) and searched in the whole-genome protein sequences of THN1. The protein sequence was blasted in NCBI, compared with other dextranases previously reported, and researched its conserved domains in the Conserved Domain Database^1^ on June 30, 2021. SignalP-5.0 Server^2^ on March 29, 2021, and ExPASy^3^ (2021.03.29) predicted the signal peptides, isoelectric points, and protein molecules of CeDex. The 3D structure of the protein was predicted by I-TASSER^4^ server on June 28, 2022. The TM-score valuing the quality of the predicted protein structure was calculated by ResQ server2 on July 23, 2022 (Yang et al., 2016). The Phylogenetic tree was established by the MEGA version 5.0 with the neighbor-joining algorithm.

### Effect of CeDex on biofilm

Biofilm mass assay was performed according to the protocol of Ren et al. (2018). The minimum biofilm inhibition and reduction concentrations (MBIC and MBRC) of *Streptococcus mutans* ATCC 25175 (American Type Culture Collection (ATCC), Manassas, VA, United States) were measured.

The activated *S. mutans* ATCC 25175 was cultured anaerobically in BHI (1% sucrose) with different dextranase activities (0, 2, 4, 6, 8, 10 U) at 37°C for 24 h. After the aspiration of the excess solution, the formed biofilms were immobilized in methanol for 15 min and dried naturally at room temperature. MBIC of *S. mutans* ATCC 25175 was measured based on the biofilm mass assay. The MBRC of *S. mutans* ATCC 25175 was valued the same way, except that the CaDex was added after the biofilm of *S. mutans* ATCC 25175 was formed.

The experimental procedures for studying the effect of dextranase on *S. mutans* biofilm referred to Ning et al. (2021). The detailed procedures were as follows: the seed solution was inoculated to BHI medium (900 μl, 1% sucrose) added with diverse concentrations of dextranase (0, 2, 4, 6, 8, and 10 U/ml) in a 24-well plate containing a sterile coverslip. After cultivating at 37°C for 24 h, the slides were gently washed with double distilled water until the nonadhesive biofilms were eliminated. The adhesive biofilms were fixed with 2.5% glutaraldehyde at 4°C for 4 h and dehydrated with 50, 70, 80, 90, and 100% alcohol gradients, each for 30 min. Finally, the samples were sprayed with gold powder and observed under SEM (Model JFC-1600, JSM-6390LA; JEOL, Tokyo, Japan).

## Results

### Isolation and identification of the dextranase-producing strain

**Figure 1:**
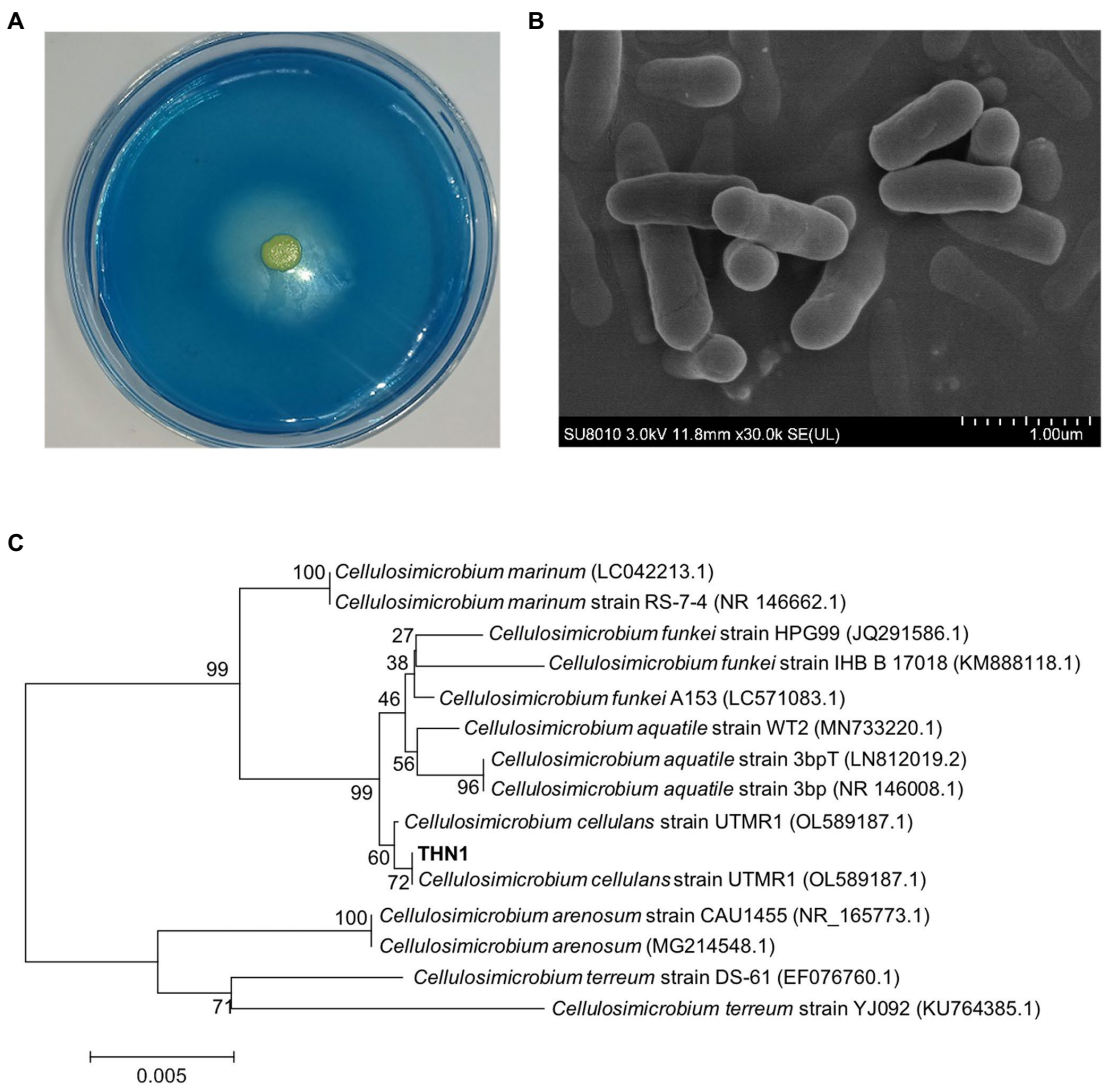
Morphological observation on the blue-dextran plate **(A)** and scanning electron micrograph **(B)**. Phylogenetic tree of strain THN1 **(C)**.

The strain THN1 (Figure 1A) was screened and showed dextranase-producing ability according to the formation of a clear zone on blue-dextran agar. THN1 was a yellow and rod-shaped bacterium by ager media and scanning electron micrograph (SEM; Figures 1A,B). Based on the alignment of 16S rDNA sequences and the results of phylogenetic analysis (Figure 1C), the THN1 strain was identified as *Cellulosimicrobium* sp. (CCTCC accession number: M2020430; China Center for Type Culture Collection, Wuhan, China).

### Purification of dextranase

**Figure 2:**
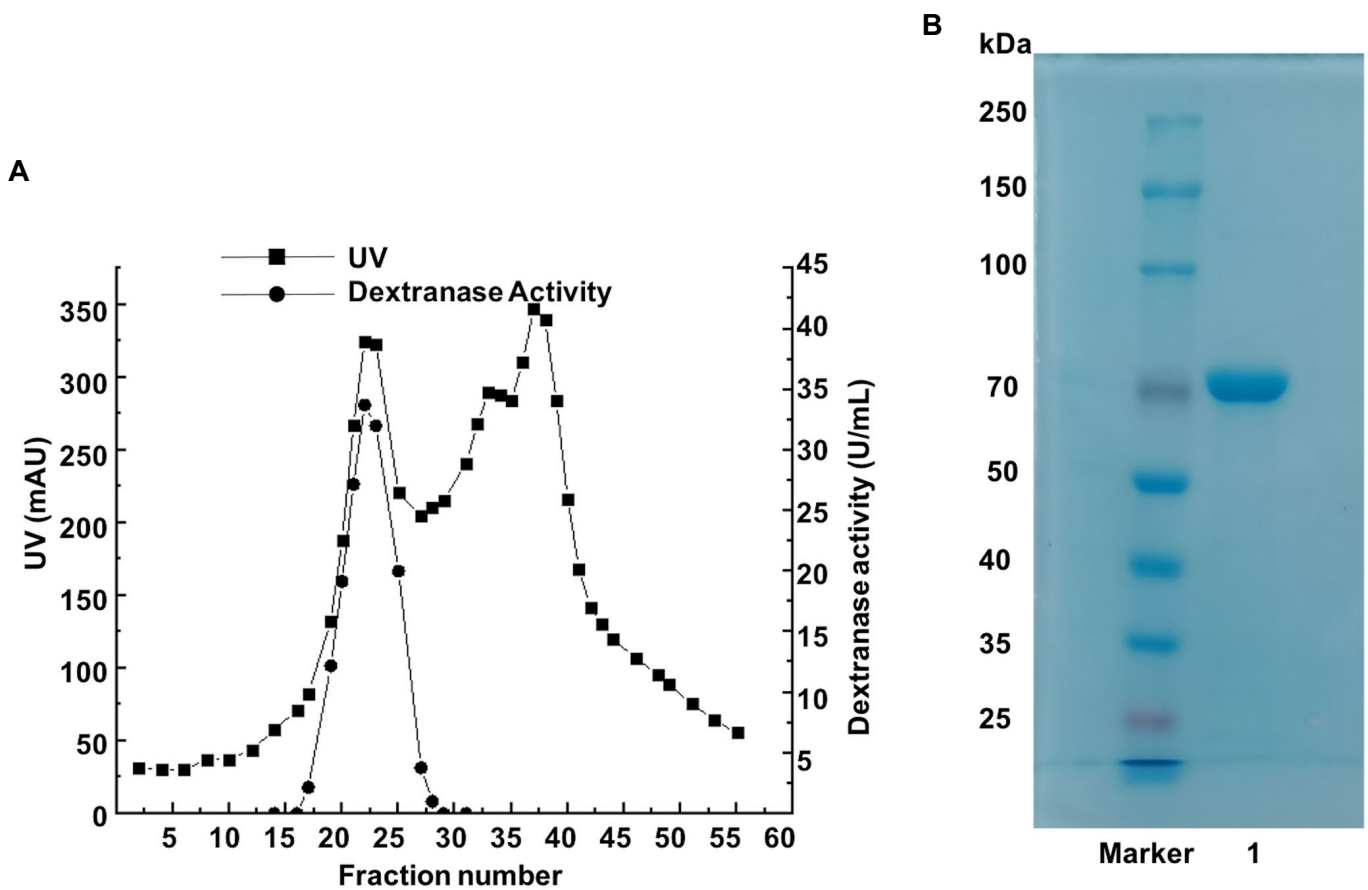
**(A)** The elution profile of the dextranase on a sepharose Q FF column. **(B)** SDS-PAGE analysis of tubes 19–23 collected from the ion-exchange chromatography. Lane Maker: protein marker. Lane 1: the purified dextranase.

By ultrafiltration, ammonium sulfate deposition, and ion exchange chromatography, the dextranase was purified to 65.5 U/mg (Table 1). During the step of ammonium sulfate precipitation, a large proportion of dextranase was precipitated and collected at 50–80% saturation. Anion exchange chromatography (sepharose Q FF) was used to optimize the purification. The eluates from tubes 19 to 23 with high dextranase activity were collected, merged and analyzed by SDS-PAGE. The result showed the purified dextranase (CeDex) was about 71 kDa (Figures 2A,B).

**Table 1:** Parameters related to purification of the dextranase (CeDex).

Purification step	Total protein (mg)	Total activity (U)	Special activity (U/mg)	Purification (-Fold)	Yield (%)
Culture broth	2048.8	3,810	1.9	1	100
30 kDa ultrafiltration	523.0	3,360	6.4	3.5	88.2
Ammonium sulfate precipitation	96.5	1,140	11.8	6.4	29.9
Ion exchange chromatography	5.1	336.8	65.5	35.2	8.8

### Enzymatic characteristics of dextranase

#### Effects of temperature and pH on dextranase activity and stability

**Figure 3:**
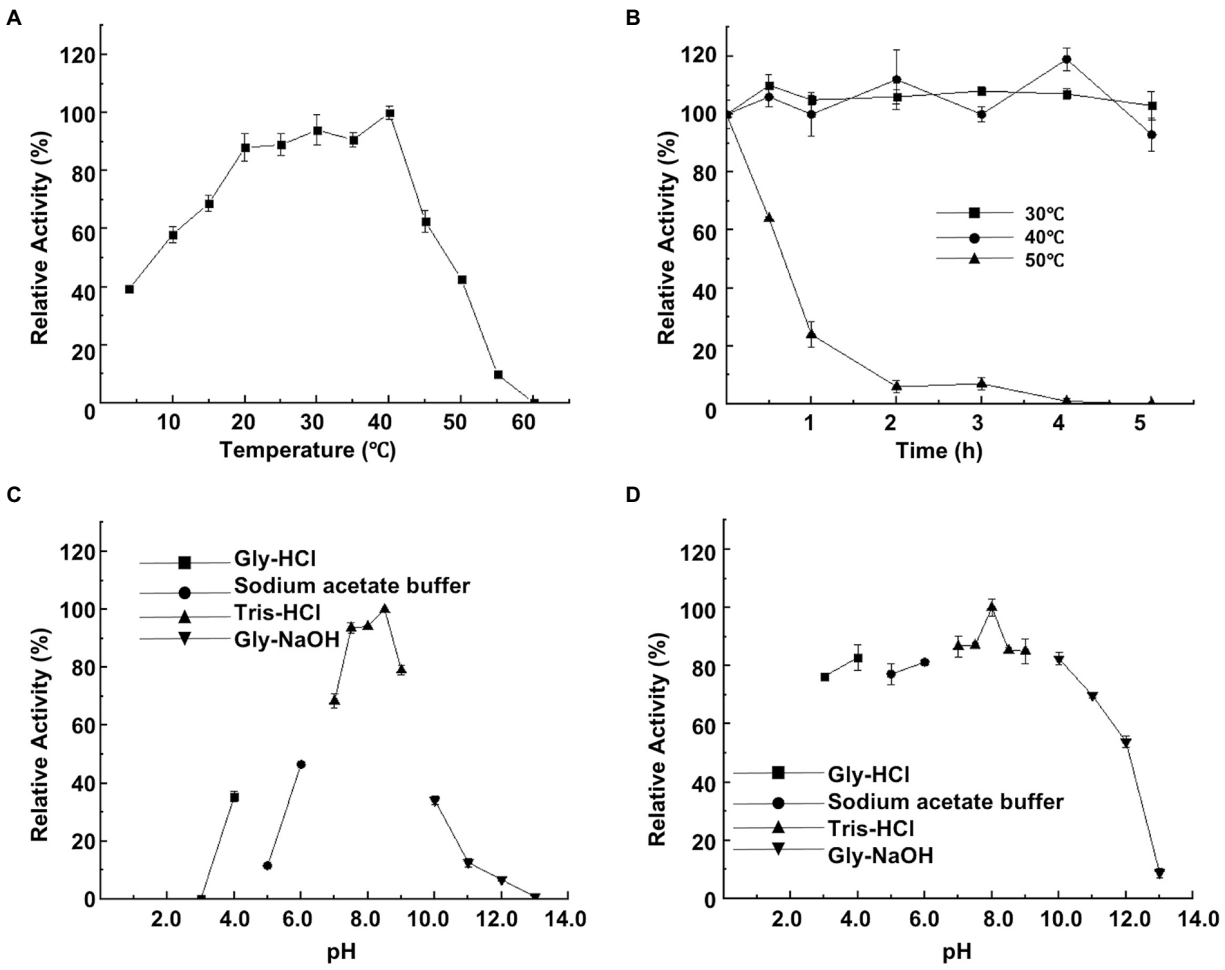
Effects of temperature and pH on CeDex activity. **(A)** optimal temperature; **(B)** thermal stability; **(C)** optimal pH; **(D)** pH stability. Error bars represent the mean ± SD (*n* = 3).

CeDex was tested at a temperature range of 4–60°C to get its apparent temperature dependence. The optimal reaction temperature was 40°C (Figure 3A). CeDex retained about 90% of the maximal activity at 20–35°C, 58% at 10°C, and 40% even at 4°C. So CeDex was a novel cold-adapted enzyme with stability in a wide range of moderate and low temperatures. Nevertheless, the CeDex activity was greatly reduced at 50°C and entirely deactivated at 60°C. The activity of CeDex at 30°C and 40°C was stabilized within 5 h (retained >80% activity); while reduced to 23% at 50°C after 1 h (Figure 3B). In addition, CeDex had the highest catalytic performance in the alkaline pH range (7–9) (Figure 3C) and excellent pH stability in pHs (3.0 to 11.0; Figure 3D).

#### Effects of metal ions, high salt, and other compounds on dextranase activity

**Figure 4:**
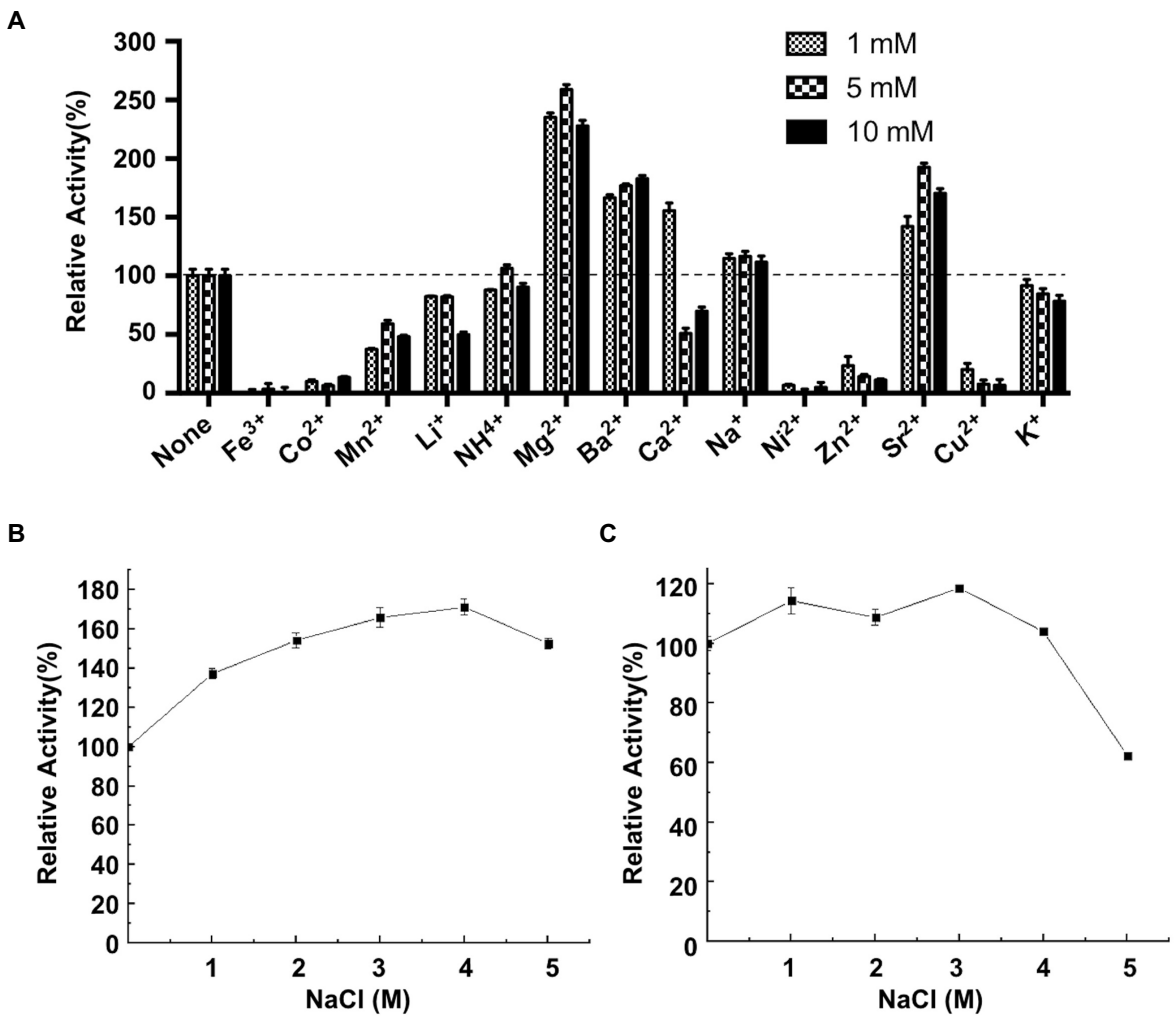
Effects of different metal ions and NaCl on CeDex activity. **(A)** metal ions; **(B)** NaCl; **(C)** NaCl stability. Error bars represent the mean ± SD (*n* = 3).

The effects of various metal ions on CeDex were researched in the optimal reaction condition (40°C, pH 8.5). Mg^2+^, Ba^2+^, Ca^2+^, Na^+^ and Sr^2+^ enhanced the CeDex activity by 1.1–2.5-fold (Figure 4A). Additionally, the relative activity of CeDex was the highest (160.5%) in 4 M NaCl solution and increased to 140–160% when the NaCl concentration was from 1 to 5 M (Figure 4B). Meanwhile, CeDex still retained 60% of its maximal activity after incubating in the 5 M NaCl solution (Figure 4C). So CeDex had excellent salt tolerance. Furthermore, the main compounds (xylitol, sodium benzoate, sodium fluoride, ethanol, sodium dodecyl sulfate, and lauric acid) in dental care products did not affect the CeDex activity significantly (Table 2).

**Table 2:** Effects of dental caries chemical treatment reagents on CeDex.

Reagents(w/v)	Relative Activity (%)
Control	100 ± 4.30
0.1% xylitol	102.19 ± 4.84
0.1% sodium fluoride	95.26 ± 4.23
0.1% sodium benzoate	99.22 ± 2.74
5% ethanol	93.02 ± 4.29
1 mM sodium dodecyl sulfate	95.69 ± 5.01
1% lanric acid	98.21 ± 3.14

#### Substrate specificity and kinetic parameters of dextranase

The substrate specificity study showed CeDex mainly hydrolyzed the dextran with α-1,6 glycosidic bonds in its main chains (Supplementary Table 1), and was less effective for soluble starch, though the starch had a small amount of α-1,6 glycosidic bonds. No activity was detected when the substrates were pullulan and chitosan, mainly constituting α-1,4 glycosidic linkages and β-1,4 glycosidic linkages, respectively. Therefore, CeDex displayed high specificity on dextran.

Table 3 displayed the enzyme kinetics of CeDex against dextran T10, T20, and T70. The *K*_m_ of CeDex for dextran T70 was 0.0991 mM, less than those of dextranases from *Arthrobacter oxydans* (4.73 mM), *Aspergillus allahabadii* X26 (14.29 mM), *Penicillium cyclopium* CICC-4022 (2.61 mM) and *Streptomyces* sp. NK458 (94.30 mM; Huang et al., 2019), demonstrating the stronger affinity of CeDex for dextran. The *K*_cat_ and *K*_cat_/*K*_m_ values of CeDex for dextran T20 were the highest, indicating that CeDex had higher catalytic efficiency for dextran T20 than T10 and T70.

**Table 3:** Enzyme kinetics of CeDex against different substrates.

Title 1	Dextran T10	Dextran T20	Dextran T70
*K*_m_ value (μM)	637.1	597.2	99.1
*V*_mac_ (μmol∙mg-1∙min-1)	36.36	46.51	31.65
*K*_cat_ (s^−1^)^1^	43.026	55.039	37.488
*K*_cat_/*K*_m_ (s^−1^∙μM^−1^)	0.068	0.92	0.378

^1^*K*_cat_ value was calculated assuming a molecular mass of 71 kDa for native dextranase.

#### Analysis of hydrolysis products

**Figure 5:**
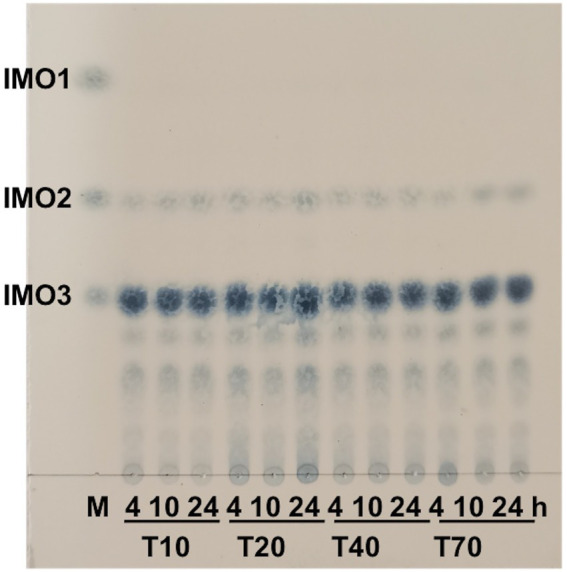
Products analysis of CeDex hydrolyzing the different molecular masses of dextran with various incubation times by TLC. M, the standard glucose (IMO1), isomaltose (IMO2) and isomaltotriose (IMO3).

The hydrolysates distribution of CeDex on different Mw dextrans (T10, T20, T40, T70) was revealed. The isomaltotetraose (G3) was the main product and higher molecular weight IMOs also existed (Figure 5). The hydrolysate distributions did not change significantly with the extension of the reaction time. The amounts of IMOs with different degrees of polymerization (DPs) in the hydrolysis products were measured by HPLC, in which isomaltotriose (G3) and DP ≥4 IMOs accounted for 58.1 and 40.7%, respectively, after 4 h of reaction. Moreover, the amounts of IMOs (DP 2–4) were increased as hydrolysis time extension with no glucose (Supplementary Table 2). Isomaltooligosaccharides are one kind of non-digestible oligosaccharides with low calorific value and can promote the growth of *Bifidobacteria* and *Lactobacillus*. Numerous isomaltooligosaccharides were produced by endodextranases and possessed broad applications in the food industry as dietary fibers and prebiotics (Sorndech, 2018). Intriguingly, the effective oligosaccharides with DPs 2–10 foreboded more applications and enormous business benefits in the food and health products (Seo et al., 2007; Liu et al., 2022).

### Nano LC–MS/MS and structure analysis of purified CeDex

**Figure 6:**
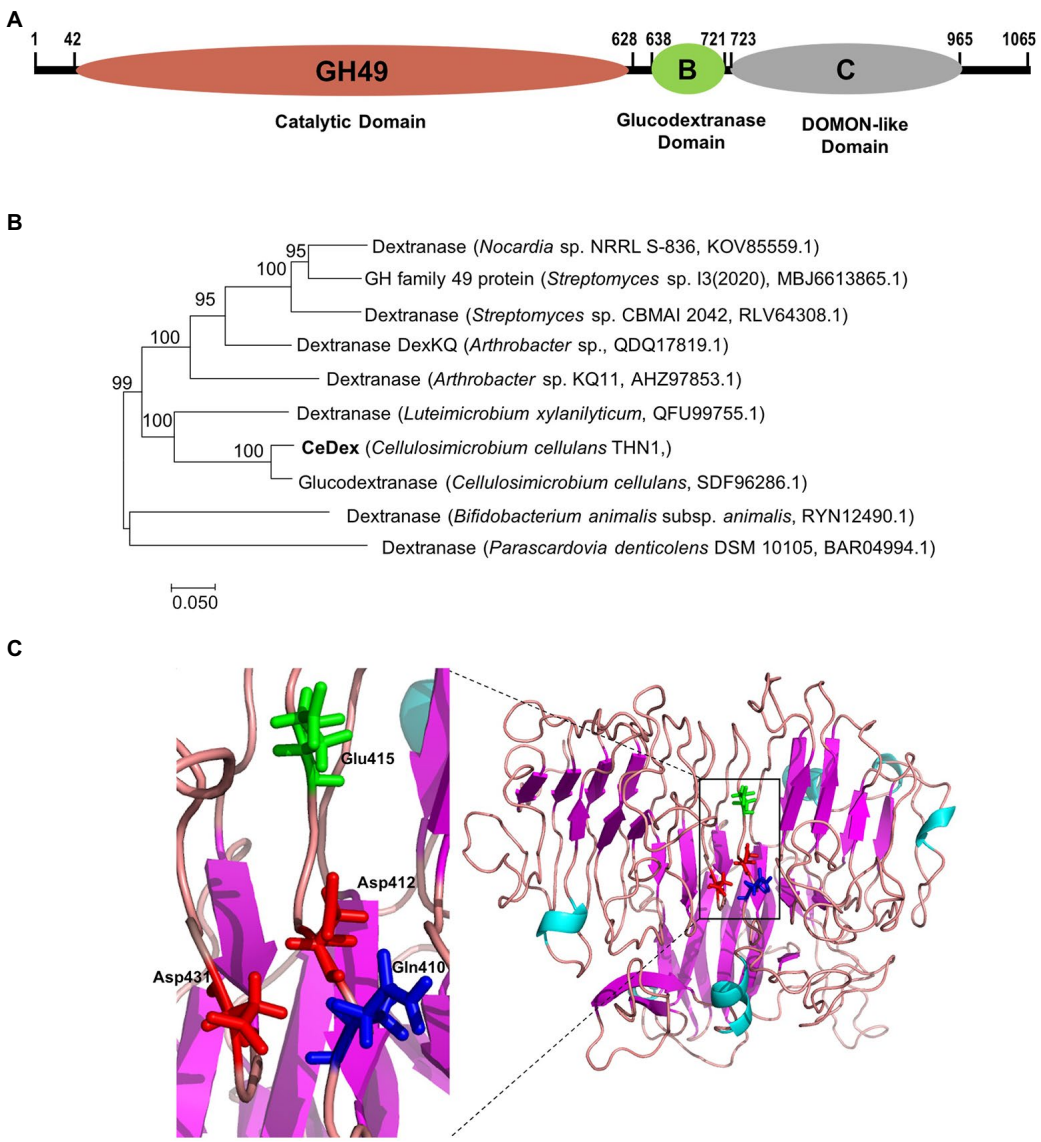
Sequence and three-dimensional structure analysis of CeDex2365. **(A)** Conserved domains analysis using the Conserved Domain Database (Lu et al., 2020). **(B)** Phylogenetic analysis of the GH49 domain of CeDex2365. **(C)** 3D structure of the GH49 domain of CeDex2365. Amino acid residues corresponding to catalysis are presented in the structure.

The peptide fragments (Supplementary Figure 1) of CeDex were obtained from nano LC–MS/MS followed by search analysis with Proteome Discoverer 1.4. The search database was the whole-genome protein sequence of THN1. The result showed the number of unique peptides of the orf2365 protein from *Cellulosimicrobium* sp. THN1 was the highest (35) and the coverage was 41.31% (Supplementary Figure 1). The orf2365 protein (CeDex2365, Genbank accession number ON856679) comprises signal peptides (M1-A23) and three primary domains -Domain of Glycosyl hydrolase family 49 (GH49, Thr^42^ to Ala^627^), Domain B of Glucodextranase (Pro^638^ to Thr^721^) and DOMON-like ligand-binding domain (Gly^724^ to Val^965^) and is identified as a multi-domain dextranase of GH49 family (Figure 6A). The peptides identified as parts of CeDex2365 by Nano LC–MS/MS were nearly all located in the GH49 domain (Supplementary Figure 1). CeDex2365 with 1,065 amino acids (AAs) has a predicted Mw of 114.49 kDa, which was utterly inconsistent with the result of the SDS-PAGE of CeDex. The N-terminal GH49 domain (A24-A637) with a predicted Mw of 68.2 kDa was close to the purified dextranase CeDex. Therefore, we deduced that CeDex was the GH49 domain of CeDex2365 (CeDex2365-49), and CeDex2365 was proteolytically cleaved after protein translation.

The phylogenetic analysis manifested CeDex2365 is closely connected with the putative glucodextranase from *Cellulosimicrobium cellulans* (Figure 6B). By submitting the sequence to I-TASSER^5^ server, the 3D structure of CeDex2365-49 was predicted (Figure 6C) using the crystal structure of Aodex from *Arthrobacter oxydans* KQ11 as a template (PDB entry: 6nzs.1. A; Ren et al., 2019). The TM-score of the predicted structure was the highest fitted score (0.61 ± 0.14; A TM-score > 0.5 indicates a model of correct topology; Zheng et al., 2021). The CeDex2365-49 consisted of a β-sandwich domain at N-terminal and a right-handed parallel β-helix domain at C-terminal (Larsson et al., 2003; Supplementary Figure 2). The signal peptide analysis by the SignalP 5.0 server showed CeDex was an extracellular protein with a signal sequence (1–23 AA of the N-terminus region; Supplementary Figure 1). Multiple sequence alignment suggested that the four predicted catalytic key residues were Asp412, Asp431, Glu415 and Gln410 (marked with stars in Supplementary Figure 2; Liu et al., 2019; Ren et al., 2019). Protein sequence alignment between CeDex and its homologs in the NCBI database indicated that CeDex exhibited high identities (92.8–99.6%) with the family 49 glycosyl hydrolases of *Cellulosimicrobium* genus (WP_257417428.1, WP_144683787.1, WP_194226287.1), which had not yet been structurally and functionally characterized. Among the reported GH49 dextranases, the catalytic GH49 domain exhibited the highest sequence identity (61.7%) with the dextranase AoDex from *Arthrobacter oxydans* KQ11 (GenBank accession number KJ571608). Then, the catalytic GH49 domain of CeDex shared 38.2 and 28.2% sequence identities with that of *Catenovulum* sp. DP03 cold-adapted dextranase (Deng et al., 2020) and *Penicillium funiculosum* dextranase (MH581385), respectively. The Gln418-Asp440 of catalytic center in CeDex was highly conserved and had high similarity with its homologs (Supplementary Figure 2).

According to recent research, one of the characteristic features of the cold-adapted and salt-tolerant enzymes was that the ratio of acidic (glutamic acid and aspartic acid) to basic (histidine, lysine, and arginine) amino acids was higher than that of thermophilic and non-salt tolerant enzymes (Ding et al., 2021). The amino acid composition of the different domains in CeDex2365 was analyzed and compared with other GH49 dextranases (Table 4). The proportion of acidic residues and basic residues in each domain of CeDex2365 was close to the cold-adapted dextranase CaDex. The net charge densities of CeDex2365 and CaDex were higher than that of AoDex and PcDex49, which did not possess cold-adaptation and salt tolerance. The high proportion of acidic amino acid in CeDex was in accord with that of the cold-adapted and salt-tolerant enzymes.

**Table 4:** Comparison of the properties and frequencies of charged amino acid residues for different domains in CeDex2365 and other dextranases.

Enzyme	CeDex2365				CaDex	Aodex	PcDex49
	Catalytic domain (GH49)	Glucodextranase Domain	DOMON-like Domain	Entire protein			
Properties	Retain ~40% of its maximum activity at 4°C				Retain ~32% of its maximum activity at 4°C	Retain <10% of its maximum activity at 10°C	Retain ~40% of its maximum activity at 35°C
Acidic residues (%)	14.3	12.5	11.1	13.0	12.7	13.3	7.8
Basic residues (%)	8.5	6.2	7.6	8.0	7.6	11.1	^7.7^
Net charge density (*z*, %)^a^	−5.8	−6.3	−3.5	−5	−5.1	−2.2	−0.1
Pro	4.9	2.3	5.8	5.0	3.7	4.1	6.1
Arg	3.6	4.2	4.4	3.9	2.2	3.0	2.1
Gly	7.2	8.1	12.0	8.8	7.9	7.1	9.9

^a^Net charge density is estimated by z = (nbasic - nacidic)/length × 100%, where nbasic and nacidic are the number of basic and acidic residues, respectively. Acidic residues include glutamic acid and aspartic acid. Basic residues include histidine, lysine, and arginine. CaDex, dextranase from Catenovulum sp. DP03; Aodex (GenBank accession KJ571608), dextranase from Arthrobacter oxydans KQ11; PcDex49 (GenBank accession MH581385), dextranase from Penicillium funiculosum.

### Effects of CeDex on biofilm

Dextran is the essential ingredient in dental plaque and protects oral bacteria within the biofilm from antimicrobials and harmful agents (Pleszczyńska et al., 2017). *S. mutans* is the primary bacteria forming dental plaque and causing dental decay (Juntarachot et al., 2020b). The minimum biofilm inhibition and reduction concentrations (MBIC and MBRC) of *S. mutans* ATCC 25175 were used to assess the effects of CeDex on biofilm (Table 5). The results showed that CeDex could inhibit biofilm formation and reduce the formed biofilm. As the concentration of CeDex increased, the inhibition and reduction rate increased. When adding 6 U/ml and 10 U/ml, CeDex could inhibit 52.3 and 91.6% of the plaque, respectively. The lowest CeDex concentration reducing biofilm by 50% was 4 U/ml, and when CeDex reached 10 U/ml, the reduction rate was up to 90.6%.

**Table 5:** The proportion of the productions of hydrolyzed dextran.

Addition of Dextranase(U/mL)	Biofilm formation inhibition rate (%)	Formed biofilm reduction rate (%)
0	0 ± 2.929	0 ± 1.488
2	9.847 ± 3.686	40.131 ± 5.628
4	36.331 ± 1.219	53.380 ± 2.455
6	52.289 ± 0.655	60.443 ± 0.175
8	71.211 ± 2.726	80.846 ± 2.348
10	91.627 ± 2.588	90.572 ± 1.455

**Figure 7:**
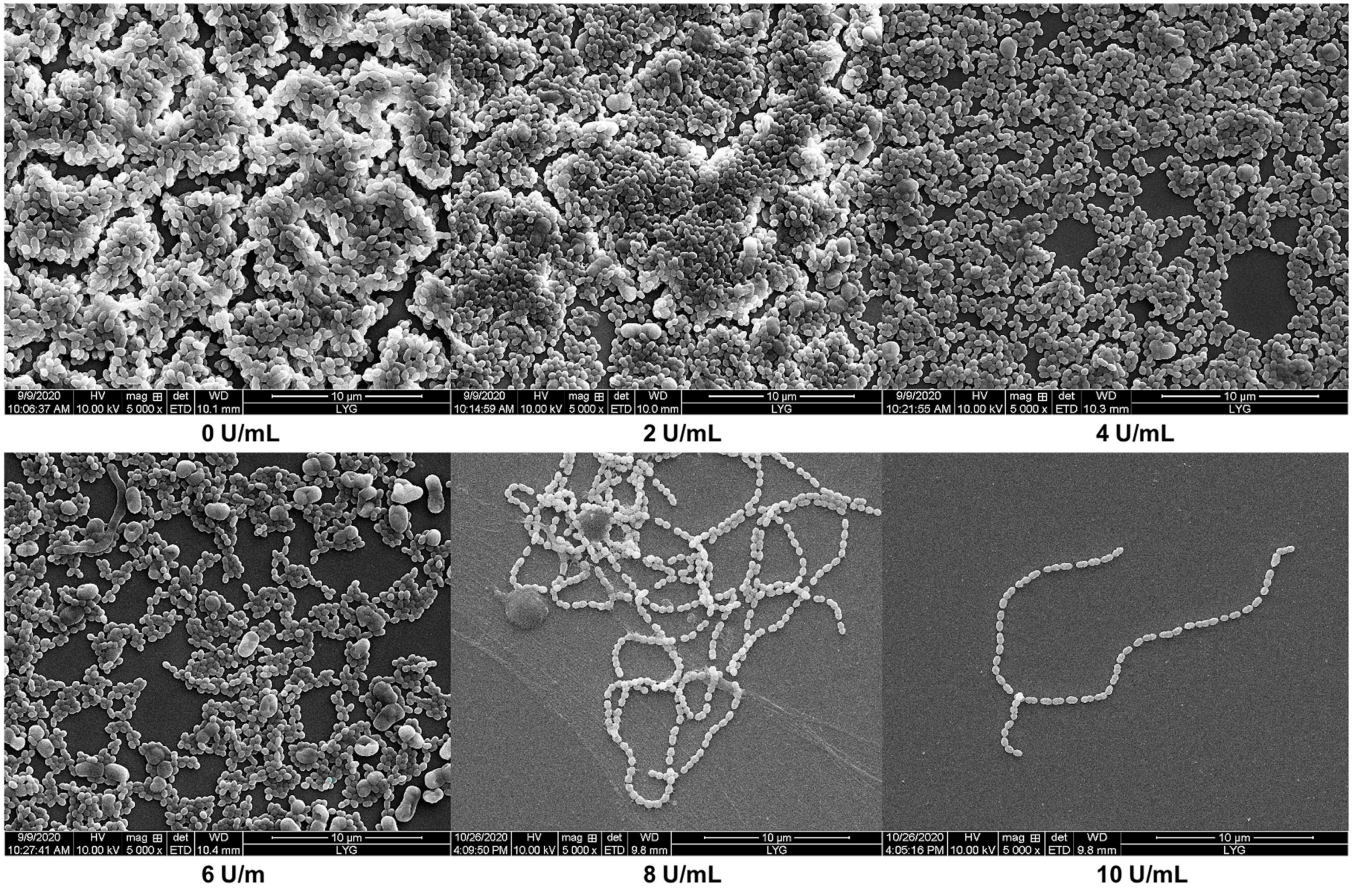
Effects of different CeDex concentrations on the biofilm formed by *S. mutans* on sterile coverslips (5000×). The presence of dextranase at the concentration of 0, 2, 4, 6, 8, and 10 U/ml. 0 U/ml presents a control with an equal amount of sterile deionized water instead of dextranase.

The morphology and biofilm of *S. mutans* were observed by scanning electron microscopy (SEM; Figure 7). The biofilm structure was tight and thick and had many channels formed by exopolysaccharides and bacteria. As the concentration of CeDex enhanced, the adhesion of *S. mutans* reduced, and the biofilm was gradually thinner and looser. The microplate crystal violet staining combined with SEM indicated that CeDex could significantly inhibit and reduce the plaque formed by *S. mutans*.

## Discussion

In this study, we purified and characterized a dextranase CeDex from a marine bacterium *Cellulosimicrobium* sp. THN1. Previous research has reported several marine strains produced dextranases, such as *Catenovulum* sp. DP03, *A. oxydans* KQ11, *Cellulosimicrobium* sp. PX02, *Bacillus aquimaris* S5 and *Catenovulum agarivorans* MNH15 (Table 6). The Mws of dextranases from marine bacteria generally range from 40 to 110 kDa and therein lie the GH49 dextranases. The GH49 conserved domain (65–71 kDa) encompasses a β sandwich composed of 190 AAs at the N-terminal and a right-handed parallel β-helix consisting of three parallel sheets at the C-terminal (Ren et al., 2018, 2019; Lai et al., 2019; Deng et al., 2020; Liu et al., 2022). The theoretical Mw of orf2365 protein was larger than CeDex. According to the results of Nano LC–MS/MS and the Mw decided by SDS-PAGE, we estimated the extracellular dextranase CeDex was the GH49 domain in the N-terminal of protein orf2365. Proteolytic cleavage of proteins was a common and irreversible post-translational modification in bacteria and was executed by endoproteases with breakups at specific sites and exoproteases with cleaving the N- and C- termini. (Forrest and Welch, 2020). Therefore, we speculated the protein orf2365 was proteolytically cleaved at C-terminal after protein translation to generate the maturation protein CeDex, and the proteolytic site needs further verification.

**Table 6:** Comparison information on the dextranases from bacteria.

Origin	MW (kDa)^1^	Classification	*T*_Opt_ and pH_Opt_	Relative activity (%)^2^ at 10/4 °C	Activator	Hydrolysis products	Reference
*Catenovulum* sp. DP03 (*E. coli* recombinant)	89.5	GH49	45°C, pH 8.0	~20 (10°C) /~8 (4 °C)	Mg^2+^, Sr^2+^	IMO3-6	Deng et al. (2020), Ren et al. (2018)
*Arthrobacter oxydans* KQ11 (*E. coli* recombinant)	66.4	GH49	55°C, pH 9.0	None	Zn^2+^, Co^2+^	IMO2, 3	Wang et al. (2016), Liu et al. (2019)
*Arthrobacter oxydans* G6-4B (native)	71	GH49	55°C, pH7.5	NR	None	IMO3	Liu et al. (2022)
*Catenovulum agarivorans* MNH15 (native)	110	NR	40°C, pH8.0	None	Sr^2+^	IMO1 ~ 3	Lai et al. (2019)
*Bacillus aquimaris* S5	~70	GH66	40°C, pH6.0	None	NR	IMO2 ~ 5	Dong et al. (2021)
*Cellulosimicrobium* sp. PX02 (native)	40	NR	40°C, pH7.5	NR	None	IMO3, IMOs	Ning et al. (2021)
*Cellulosimicrobium cellulans* THN1 (native)	~71	GH49	40°C, pH8.5	59 (10°C) /40 (4 °C)	Mg^2+^, Na^+^, Ba^2+^, Ca^2+^, Sr^2+^	IMO3, IMOs	This study

NR, not reported.

^1^Molecular mass of the monomer calculated from the amino acid sequence or determined by SDS-PAGE.

^2^Relative activity obtained at the temperature indicated in brackets was calculated as the percentage of the activity at *T*_Opt_.

To adapt to marine extreme environments, marine microorganisms produce cold-adapted and/or salt-tolerant enzymes, which have a promising prospect in applied biotechnology and basic research (Ding et al., 2021). The dextranase (CeDex) from the marine bacterium, *Cellulosimicrobium* sp. THN1, exhibited cold-adaptation, salt tolerance, excellent pH stability and sufficient thermostability. To the best of our knowledge, the dextranase CaDex from *Catenovulum* sp. DP03 was the only cold-adapted one and no salt-dextranase had been reported. CeDex kept more than 90% of the highest activities in a broad temperature range (20–40°C) and more than 58% at 10–20°C, which was distinguished from the conventional fungal dextranases (Wang et al., 2022). CaDex from *Catenovulum* sp. DP03 remained more than 70% of the highest activity at 30–50°C, about 40% at 20°C and 20% at 10°C (Table 6). In short, the CeDex activity at low temperatures (4–20°C) was higher than CaDex from DP03, and the cold-adapted property of CeDex was superior to other marine dextranases. Even though the fungal dextranases had higher enzymatic activity than bacterial dextranases, nearly all fungi dextranases were acidic and sensitive to alkaline in varying degrees (Khalikova et al., 2005). Previous research had demonstrated that alkaline dextranases had more advantages in the prevention and treatment of dental caries than acid dextranases because the alkaline mouthwash was more protective than enamel against acidic (Ren et al., 2018; Lai et al., 2019). Meanwhile, alkali production of oral bacteria plays an important part in oral ecology, pH homeostasis, and inhibiting the formation of dental caries (Huang et al., 2017). CeDex had enzyme activities in neutral and alkaline conditions (pH 7–9) and maintained good stability. So, CeDex was also an alkaline dextranase and has the potential to be a dental caries reagent. In recent research, marine dextranases were reported to effectively sweep away dental plaque, for example, dextranases from *Cellulosimicrobium* sp. PX02, *Arthrobacter oxydans* KQ11, *Catenovulum agarivorans* MNH15, *Catenovulum* sp. DP03 and *Bacillus aquimaris* S5 (Wang et al., 2016; Lai et al., 2019; Deng et al., 2020, 2021). In order for the inhibition rate of dental plaque biofilms to be 90%, the concentration of PX02 dextranase was at least 15 U/ml (Ning et al., 2021), AoDex and MNH15 dextranase needed to be 6–7 U/ml (Wang et al., 2016; Lai et al., 2019), the dextranase from *Catenovulum* sp. DP03 should be 8 U/ml (Dong et al., 2021). When the dextranase of *Bacillus aquimaris* S5 was 8 U/ml, the inhibition rate was 80% (Dong et al., 2021). Though AoDex had a highly efficient effect on inhibiting the biofilm formed by *S. mutans*, the biofilm reduction ratio was only 65% (6 U/ml; Wang et al., 2016). In addition, SDS had an effective inhibiting effect on the enzymatic activity of MNH15 dextranase and AoDex (Ren et al., 2018; Lai et al., 2019). Intriguingly, SDS and other compounds in dental care products did not suppress CeDex activity distinctly. Therefore, in consideration of the good preventing and treating effects on the biofilm of *S. mutans* (MBRC_90_ and MBIC_90_ were 10 U/ml) and excellent stability, the cold and alkalic CeDex will be more suitable for the exploitation of novel therapeutic agents for dental caries.

There has not been a clear definition of salt-tolerant enzymes until now. According to published research, they were a kind of enzyme with activity and/or stability under high NaCl concentrations. Different types of salt-tolerant enzymes had different tolerance ranges, for example, the NaCl concentration of salt-tolerant xylanase was 0.5–6.0 M (Cao et al., 2021), the NaCl concentration of halotolerant protease was 4–5 M (Mokashe et al., 2018), and the highest NaCl concentration of salt-tolerant esterase was 5 M (Wu et al., 2015; Jin et al., 2019). To our knowledge, no salt-tolerant dextranase has been reported to date. The highest NaCl concentration of retaining CeDex’s catalytic activities was 4 M and CeDex also had enzymatic activity in low NaCl conditions (~0.2 M; Figure 4B), which was different from most halophilic enzymes relying on high concentrations of NaCl to maintain their activities stably (Zhang et al., 2018). In addition, CeDex exhibited favorable stability in a high NaCl concentration (1–4 M NaCl). Salt-tolerant enzymes have great significance in biotechnology and research on the extreme environmental adaptation of microorganisms. Furthermore, halophilic enzymes with stable or stimulative activity in high-salt conditions could be available for the high-salt process (Schreck and Grunden, 2014; Yang et al., 2020). In the sugarcane industry, dextranase is mainly used to remove dextran, which pollutes the sugar juice and reduces the yield of sucrose (Bashari et al., 2019). Sugarcane juice contains cations like K^+^, Ca^2+^, Mg^2+^ and anions like Cl^−^, PO_4_^3−^ and SO_3_^2−^, which inhibited dextranase activity (Purushe et al., 2012; Wang et al., 2016; Ren et al., 2018; Lai et al., 2019). The CeDex activity was significantly enhanced by Na^+^, Mg^2+^, Ba^2+^, Ca^2+^, and Sr^2+^ (1 mM) and K^+^, Li^+^, and NH^4+^ did not reduce the CeDex activity. In short, the excellent thermostability, salt tolerance, and metal ions stability made CeDex adaptable for the sugarcane industry (Purushe et al., 2012; Liu et al., 2022).

## Conclusion

Our work revealed a new endo-type dextranase belonging to the GH49 family from a marine bacterium *Cellulosimicrobium* sp. THN1. CeDex was a cold-adapted dextranase with high activities at medium and low temperatures (10–40°C) and had excellent thermal and pH stability. Notably, CeDex was the first dextranase with activity and/or stability under a NaCl concentration of 1–4 M. CeDex was stimulated by Mg^2+^, Ca^2+^, and K^+^ ions in sugarcane juice, and effectively inhibit and/or eliminate the *S. mutans* biofilm unimpeded by some reagents of dental care products. Therefore, CeDex has great potential applications in the sugarcane industry and in manufacturing oral hygiene products to remove dental plaque.

## Data availability statement

The original contributions presented in the study are included in the article/Supplementary Material, further inquiries can be directed to the corresponding authors.

## Author contributions

LX and YZ designed the experiments. YZ performed the experiments. LX, NL, ZWe, and ZWa analyzed the data. LX wrote the manuscript. SW and YW reviewed the manuscript. All authors contributed to the article and approved the submitted version.

## Funding

This work was funded by research grants from the National Natural Science Foundation of China (grant no. 32172154), the Jiangsu Planned Projects for Postdoctoral Research Funds (2019 K215), the Research Start-up Fund of Jiangsu Ocean University (KQ17019), the Natural Science Foundation of Jiangsu Higher Education Institutions of China (grants no. 22KJB180015), the Natural Science Foundation of Jiangsu Province (grants no. BK20201028), the Postdoctoral Research Funds of Lianyungang.

## Conflict of interest

The authors declare that the research was conducted in the absence of any commercial or financial relationships that could be construed as a potential conflict of interest.

## Publisher’s note

All claims expressed in this article are solely those of the authors and do not necessarily represent those of their affiliated organizations, or those of the publisher, the editors and the reviewers. Any product that may be evaluated in this article, or claim that may be made by its manufacturer, is not guaranteed or endorsed by the publisher.

## Footnotes

^1^ https://www.ncbi.nlm.nih.gov/Structure/cdd/wrpsb.cgi

^2^ http://www.cbs.dtu.dk/services/SignalP/

^3^ https://prosite.expasy.org/

^4^ https://zhanggroup.org/I-TASSER/

^5^ https://zhanggroup.org/I-TASSER/
