# Supplementary material for: Purification and characterization of cold-adapted and salt-tolerant dextranase from Cellulosimicrobium sp. THN1 and its potential application for treatment of dental plaque
Source: Front Microbiol. 2022 Nov 11;13:1012957. doi: 10.3389/fmicb.2022.1012957 (PMC9691899; doi:10.3389/fmicb.2022.1012957)
Supplement: Supplementary file 1 [file Data_Sheet_1.docx]

Supplementary Material

# Supplementary Figures and Tables

## Supplementary Figures


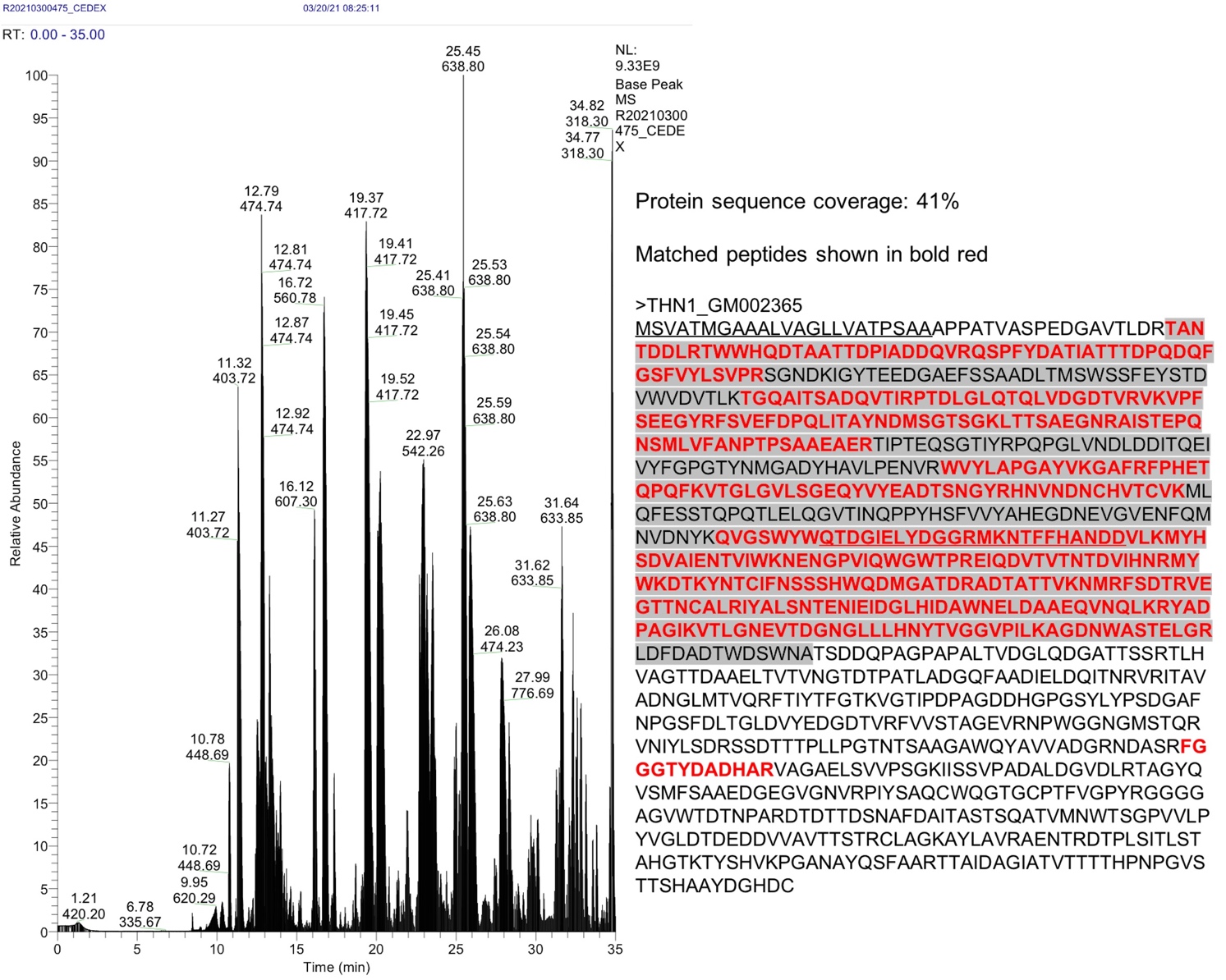


**Supplementary Figure 1.** Nano LC-MS/MS spectrum of tryptic digested peptides of CeDex of *C. cellulans* THN1. Matched peptides shown blod red, the GH49 domain was marked by gray shadow.


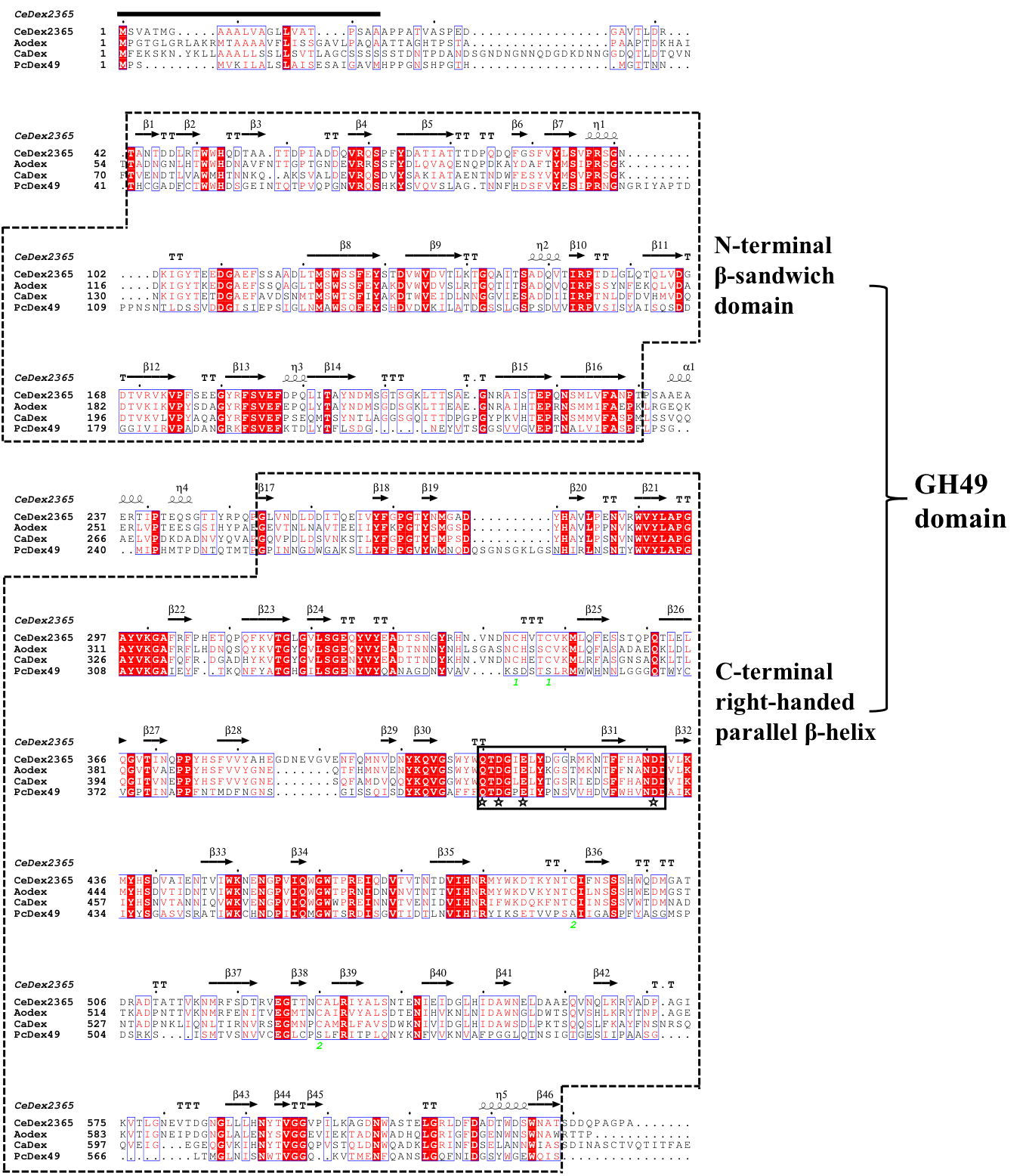


**Supplementary Figure 2.** Structure-based amino acid alignment of CeDex with its structural homologs. Secondary structure elements (helices with squiggles, β-strands with arrows, β-turns with TT letters, and 3_10_-helix with η) of CeDex are presented on top. Sequences (GenBank accession numbers or reference): CeDex2365, *Cellulosimicrobium* sp. THN1 dextranase (ON856679); Aodex, *Arthrobacter oxydans* KQ11 dextranase (KJ571608); CaDex, *Catenovulum* sp. DP03 (Deng et al., 2020); PcDex49, *Penicillium funiculosum* dextranase (MH581385). The predicted signal peptide is labeled by a black bar and the conserved domain is outlined by dotted line. The conserved amino acids are shown in shaded red boxes (white letters). Residues with >70% similarity, based on physicochemical properties, are shown in white boxes (red letters). Cysteine residues that form disulfide bonds are labeled with green numbers. Four predicted catalytic key residues were marked with stars. This figure was created with ESpript 3.0.

## Supplementary Tables

**Supplementary Table 1.** Effects of CeDex on diverse carbohydrates.

| **Substrate** | **Main Linkages** | **Relative Activity** |
| --- | --- | --- |
| Dextran T10 | α-1,6 | 100±3.08 |
| Dextran T20 | α-1,6 | 98.22±0.56 |
| Dextran T40 | α-1,6 | 95.31±1.58 |
| Dextran T70 | α-1,6 | 93.33±3.76 |
| Dextran T500 | α-1,6 | 82.85±2.26 |
| Dextran T2000 | α-1,6 | 85.6±0 |
| Pullulan | α-1,4，α-1,6 | 0.00 |
| Soluble starch | α-1,4，α-1,6 | 11.21±0.41 |
| Chitosan | β-1,4 | 0.00 |

**Supplementary Table 2.** The proportion of the productions of hydrolyzed dextran T20.

| **Time of Hydrolysis (h)** | The proportion Hydrolysis Productions (%) | | | |
| --- | --- | --- | --- | --- |
|  | Isomaltose | Isomaltotriose | Isomaltotetraose | Long-chain IOMs (DP≥4) |
| 4 | 1.143 | 58.143 | 8.308 | 32.406 |
| 10 | 1.708 | 57.342 | 8.507 | 32.443 |
| 24 | 2.263 | 62.035 | 9.529 | 26.173 |
